# Surface Modification of Synthetic Zeolites with Ca and HDTMA Compounds with Determination of Their Phytoavailability and Comparison of CEC and AEC Parameters

**DOI:** 10.3390/ma15124083

**Published:** 2022-06-08

**Authors:** Michał Łach, Agnieszka Grela, Kinga Pławecka, Martin Duarte Guigou, Janusz Mikuła, Norbert Komar, Tomasz Bajda, Kinga Korniejenko

**Affiliations:** 1 Faculty of Material Engineering and Physics, Cracow University of Technology, Jana Pawła II 37, 31-864 Cracow, Poland; michal.lach@pk.edu.pl (M.Ł.); kinga.plawecka@pk.edu.pl (K.P.); janusz.mikula@pk.edu.pl (J.M.); 2 Faculty of Environmental and Power Engineering, Cracow University of Technology, Warszawska 24, 30-155 Cracow, Poland; agrela@pk.edu.pl; 3 Facultad de Ingeniería y Tecnologías, Universidad Católica del Uruguay, B de Octubre 2738, Montevideo 11600, Uruguay; martin.duarte@ucu.edu.uy; 4 Ekologia Przedsiębiorczość Innowacje Spółka z o.o., Kühna 17, 42-256 Olsztyn, Poland; norbert.komar@epi.czest.pl; 5 Faculty of Geology, Geophysics and Environmental Protection, AGH University of Science and Technology, Adama Mickiewicza 30, 30-059 Cracow, Poland; bajda@agh.edu.pl

**Keywords:** synthetic zeolite, surface modification, phytoavailability

## Abstract

Zeolites obtained from fly ash are characterized by very good anion- and cation-exchange properties and a developed porous structure. This paper presents the results of surface modification studies of synthetic zeolites obtained from calcined coal shale (clay materials). Calcium compounds and hexadecyltrimethylammonium bromide (HDTMA) were used as modifying substances. The characteristics of the raw material and the zeolite obtained as a result of its synthesis are presented. The surface modification method is described. Furthermore, the results of sorption and desorption of NO_3_, PO_4_, and SO_4_ from raw and modified samples are presented. The results of anion- and cation-exchange capacities for other zeolite types were also compared. Modification of the materials with Ca ions and HDTMA surfactant only improved the sorption of sulfates. The 90% desorption of nitrates, phosphates, and sulphates from the zeolite material without modification indicates a good release capacity of these compounds and their potential use as fertilizer additives.

## 1. Introduction

Zeolites are defined as tectosilicates, three-dimensional inorganic polymers. They are made up of SiO_4_ tetrahedrons, some of which can be replaced by AlO_4_ [1,2,3]. In the composition of the zeolites, in addition to the crystal network and ion exchange cations, there is also zeolite water, which is continuously released when the zeolites are heated to 400 °C, which does not change the shape of the zeolite crystals [4,5].

Because of the specific internal construction of zeolites, which gives them various beneficial physical and chemical properties, it is possible to use these materials in industrial processes. Not only single atoms, but also molecules of chemical compounds can diffuse and penetrate into the interior of zeolites because the size of the channels in zeolites is large [6,7,8,9].

On the basis of unique structural properties, framework types of zeolites are defined by the International Zeolite Association Structural Commission (IZA-SC) [3]. The zeolites can be divided by the molar ratio of Si/Al in the crystalline skeleton. In this classification, low-silicate zeolites Si/Al ≤ 2, medium-silicate zeolites 2 < Si/Al ≤ 5, and high-silicate zeolites 5 < Si/Al can be distinguished [10,11].

Zeolites with low silica content Si/Al < 2 have good ion exchange ability. They can be used to soften water [12], remove ammonium [13], and remove heavy metals, e.g., zinc [14], nickel [15], copper [16], and cadmium [17]. High-silicon zeolites with a Si/Al ratio of several thousand are produced industrially [18,19].

The first natural zeolite analogue was obtained by synthesis in 1948 by Barrer [20]. The synthesis of zeolites is generally carried out under hydrothermal conditions in an alkaline environment [21,22,23]. The work on the synthesis of zeolites from fly ash was started by Professor Querol’s team in the 1980s. At that time, a similarity was found between the chemical and mineral composition of fly ashes and volcanic ash, which were formed during volcanic eruptions, from which natural zeolites were formed [24,25]. In the case of fly ash, the synthesis can be carried out using the hydrothermal method, in which the ash is directly subjected to alkaline solutions of NaOH or KOH at a given temperature and pressure for a given period of time, which is the most commonly used method [26]. The main source of the elements Si and Al is the aluminosilicate phase, but these elements may also come from the mineral phases (quartz and mullite) contained in ash. The largest share in the zeoliticization process is represented by the amorphous aluminosilicate glaze, which dissolves quickly in an alkaline environment [27].

Synthetic zeolites have hydrophobic properties, due to which they absorb organic molecules and have hydrophilic properties, which also make them effective as gas dryers [28,29]. The ion-exchangeable properties are also a characteristic feature of zeolites and are used to neutralize wastewater and treat heavy metals and radioactive elements in water [30,31,32,33,34].

The negative charge of the crystal lattice of zeolites, caused by heterovalent substitutions of aluminum for silicon in the structure of minerals, predisposes them to function as natural cationic adsorbents. Depending on the requirements of various industries, various exchangeable cations can be introduced into the structure of zeolites and the surface properties of minerals may be freely modified [35].

**Figure 1:**
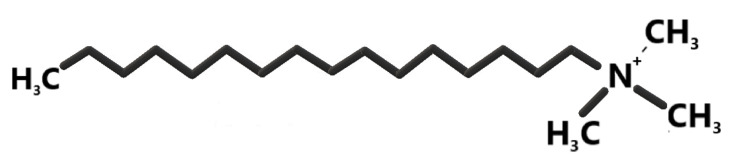
Structural structure diagram of the HDTMA molecule.

The use of zeolite phases in the adsorption processes of anionic forms or hydrophobic organic compounds is conditioned by a previous modification of mineral surface properties. Studies in the literature show that long-chain organic compounds, represented by HDTMA (Figure 1). The zeolites modified with organic compounds are called organo-zeolites [36,37,38].

The HDTMA molecule has a characteristic amphiphilic structure, manifested by the division of the molecule into two segments with different properties. There is a hydrophobic part, formed from an aliphatic chain containing 16 carbon atoms, insoluble in polar liquids and connected to it by a covalent bond, and a hydrophilic head with lipophobic properties, built from a nitrogen atom surrounded by three methyl groups. The head of the molecule is endowed with a permanent positive charge, located at the nitrogen molecule. Nitrogen is pentavalent and participates in the formation of only four bonds, which results in the generation of an excess positive charge. This charge is balanced by the negative charge of the counter ion, which in most cases is a Br- or Cl- atom [37]. A consequence of this structure is the ability of the surfactants to align themselves in an oriented manner on the surface of the phase contact and reduce surface tensions.

The sorption of surfactant compounds on the zeolite surface involves ion exchange processes and hydrophobic interactions and presents a different course depending on the concentration of surfactant in solution [39].

**Figure 2:**
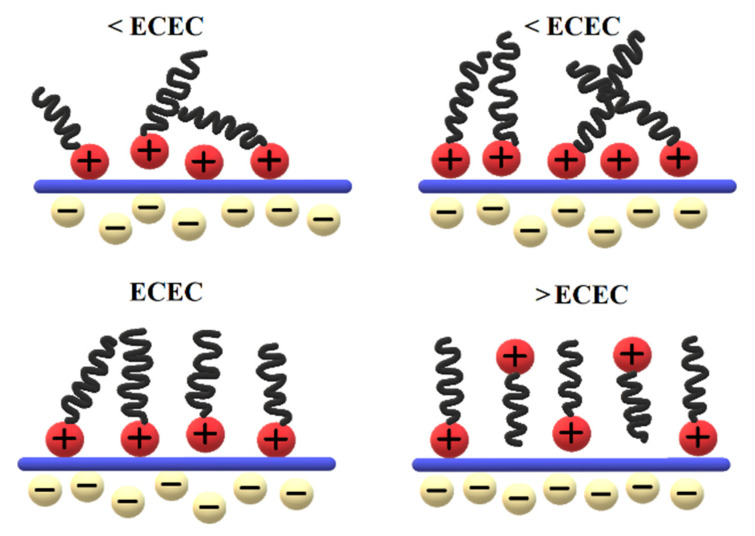
Mechanism of surfactant sorption on the zeolite surface.

The cationic surfactant represented in this work by HDTMA is too large to penetrate the zeolite structure, therefore only surface interaction is observed [40]. If an amount of surfactant less than the external cation exchange capacity (ECEC) is introduced, organic cations adsorbed with positively charged heads on the mineral surface form a monomeric layer. As the surfactant is subsidized, a thickening of the structure is observed. The formation of the second, oppositely positioned layer is a result of the organic cations’ tendency to form micelles. When the ECEC value is exceeded, a second monolayer is attached to the first one on electrostatic interactions between hydrocarbon chains. The mechanism of sorption of cationic surfactants is presented in Figure 2. The second layer with hydrophobic properties is capable of adsorbing anions [37,41]. In fact, the first layer already shows some sorption properties toward anions, as incomplete charges can be formed and an excess of positive charges can be generated at some places on the zeolite surface. The adsorption capacity towards anions presented by the surfactant monolayer is explained by the heterogeneity of the charge density on the zeolite surface [36,37,38,42,43].

**Figure 3:**
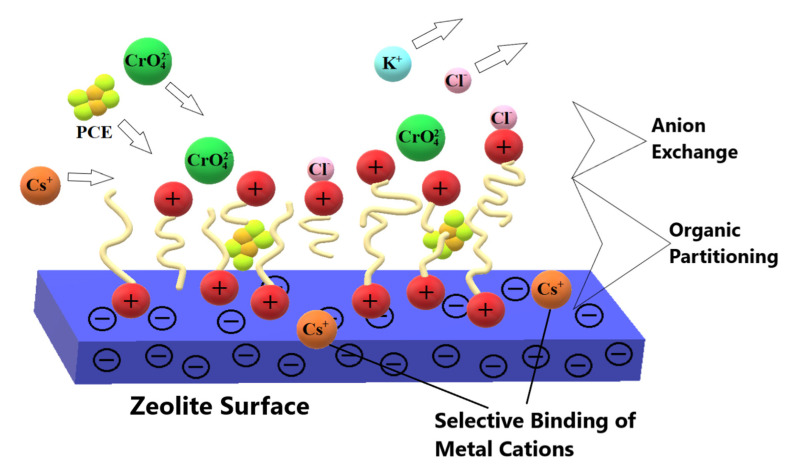
Mechanism of anion sorption on the organo-zeolite surface.

The organo-zeolite retains a low affinity for transition metal cations, which can bind to the mineral surface by ion exchange. Moreover, modification of the mineral with long-chain organic compounds leads to the formation of a zone responsible for the sorption of molecules of apolar alkylaromatic compounds. Because of its various sorption capacities, organic zeolite is a mineral with a wide range of applications [44]. The mechanism of anion sorption in organo-zeolite is shown in Figure 3.

The aim of this study was to modify the surface of synthetic zeolite materials with Ca and HDTMA compounds, and to determine their phytoavailability by comparing cation exchange capacity (CEC) and anion exchange capacity (AEC) parameters. Determining the degree of phytoprotection allows the bioavailability of various types of ingredients present in the base material.

Coal shale, which is a waste material, was used to synthesize zeolite materials. Scanning electron microscopy (SEM), Brunauer, Emmett, and Teller (BET), and X-ray diffraction (XRD) were used to characterize the zeolite materials and their modifications. The novelty of this work lies in the use of previously calcined coal shale for the synthesis of zeolite materials, which even without modification showed very high values of sorbed nitrates, phosphates, and sulphates. Modification of these materials with Ca ions and HDTMA surfactant only improved sulphate sorption. The desorption of nitrates, phosphates, and sulphates from the zeolite material without modification amounting to 90% indicates a good release capacity of these compounds and their potential use as fertilizer additives. Furthermore, the experimental studies carried out can be very supportive of conducting further research on a larger scale.

Sorption materials can also be used in foamed geopolymers as a functional additive. It was confirmed that coal shale can also be a precursor for the production of foamed geopolymers [44], and the addition of zeolites can make them applicable as filters, for example, for cleaning water reservoirs. Any research in this area is extremely interesting and useful.

## 2. Materials and Methods

### 2.1. Materials and Sample Preparation

**Figure 4:**
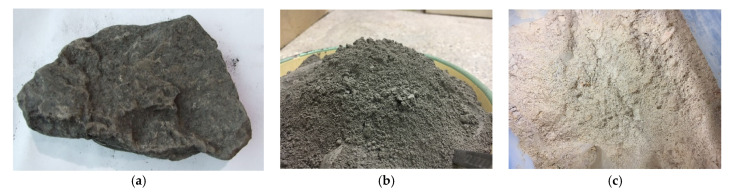
Coal shale used in the study: (**a**) in raw form; (**b**) after mechanical treatment—crushing and grinding; (**c**) after calcination.

Samples for the study were collected from a Polish coal mine, Ruch Rydułtowy (Rydułtowy, Silesia Province, Poland) (Figure 4a). The material was first crushed in a jaw crusher and then ground in a ZM200 RETSCH (Retsch, Hann, Germany) ultracentrifugal mill, screen (0.040 mm) (Figure 4b). Based on the thermal analysis measurements previously described by the authors, it was determined that the optimal temperature for the calcination process of the raw materials studied was 750 °C [45] (Figure 4c). 

Due to the desirable petrographic composition characterized by a high clay content (Table 1), this material is an attractive raw material for the neolithization process.

The synthesis of the AS sample was carried out in cylindrical vessels made of polypropylene with a volume of 1 dm^3^, and the ground slate after calcination was mixed with the activator sodium hydroxide NaOH (purity > 98%). The solid to activator was 1.5 g/5 g, which means that for every 1.5 g of material after calcination, there was 5.0 g of NaOH. The synthesis was carried out at 80 °C for 24 h. These synthesis conditions were chosen based on results reported in the literature [46,47].

Table 2 shows the description of the samples.

### 2.2. Methodology

The characterization of the RM and AS samples was performed using the following techniques. X-ray diffraction (XRD) data were obtained using an X-ray diffractometer (RIGAKU XRD, XD, RIGAKU, Tokyo, Japan) from 3° to 70° (2θ), with scanning (2θ), at a scanning rate of 0.05° per minute. Samples with particle sizes smaller than 200 μm were tested. Scanning electron microscopy (SEM, JEOL JSM 820, JEOL, Peabody, MA, USA) analysis was used to investigate the surface morphology of the RM and AS samples. The surface and textural properties of the material before and after modifications were studied by N_2_ adsorption-desorption isotherms using Micromeritics ASAP 2020 instrument.

### 2.3. Modification of the Material after the Synthesis (AS)

#### 2.3.1. Modification by Sorption of Ca Ions

First, 4 g of the sample ground in an agate mortar was poured into 40 mL of CaCl_2_ solution of 0.25 M. The mixture was shaken for 24 h and centrifuged for 5 min at 4500 rpm, after which the solution was decanted from the precipitate. The procedure was repeated 3 times. The obtained sample was washed 8 times with redistilled water and then dried at 60 °C for 48 h. To determine the amount of Ca^2+^ ions introduced into the positions of the synthetic zeolite, an experiment was carried out to desorb them using 1 M NH_4_Cl. For this purpose, 1 g of the modified sample was treated with 50 cm^3^ of 1 M NH_4_Cl. After 2 h of shaking, the sample was centrifuged (10 min, 4500 rpm) and the Ca concentration in the solution was determined. At the same time, the same procedure was applied to the unmodified sample to determine the amount of Ca^2+^. 

#### 2.3.2. Modification by HDTMA

First, 3 g of the sample ground in an agate mortar was poured over 50 mL of redistilled water and stirred on a magnetic stirrer together with heating for 1 h. Then, 115 mg of HDTMA dissolved in 50 mL of redistilled water was added. The 115 mg HDTMA added to 3 g of sample corresponds to the CEC value of the sample (13.57 meq HDTMA/100 g sample). The solution was stirred in a magnetic stirrer along with heating for 2 h. The resulting mixture was filtered through a filter and then dried at 60 °C for 48 h.

### 2.4. Methodology for Determining the Cation Exchange Capacity (CEC) and the Anion Exchange Capacity (AEC)

#### 2.4.1. Determination of the Cation Exchange Capacity of CECs by Sorption of the Quaternary Ammonium Salt Hexadecyltrimethylammonium Bromide (HDTMA)

First, 1 g of the sample grated in an agate mortar was poured over 100 mL of redistilled water and stirred on a magnetic stirrer together with heating for 3 h. Then, 1.5 g of HDTMA dissolved in 50 mL of redistilled water was added. The mixed solutions were stirred on a magnetic stirrer together with heating for 24 h. After this time, the stirring was turned off for 1 h, the solution was poured from the precipitate, and then another portion of 1.5 g of HDTMA of the same concentration as before was added and the solution was stirred again for 5 h. After being cooled, the sample was filtered through a filter. The resulting material was washed with hot water on a filter until the chloride reaction disappeared. Then, the sample was washed with hot ethanol and dried at 60 °C for 24 h.

The CHN content of the sample was determined using an automatic ElementarVarioEL III analyzer.

#### 2.4.2. Determination of the Cation Exchange Capacity (CEC) by Concentration of NH_4_ Ions

First, 100 ± 1 mg samples of material were placed in 7 mL polypropylene tubes and flooded with 5 mL of 1 M ammonium acetate CH_3_COONH_4_. The suspension of the sample in the solution was shaken on a shaker (150 rpm) for 2 h, removed from the shaker, and left for 96 h to allow the ion exchange process of NH_4_^+^ ions for natural ions that occupy the ion exchange positions. After this time, the samples were centrifuged for 20 min at 14,000 rpm and then the solution was decanted from the sediment. This process was repeated three more times. After the final stage of shaking, centrifuging, and decanting, 5 cm^3^ of ethanol was added to the sediment in the tube, shaken on the shaker for 5 min, centrifuged, and alcohol decanted. This procedure was repeated two more times. The purpose of using ethanol was to wash out the ammonium ions that were not bound at the ion exchange positions. After this step, the sample was flooded with 5 cm^3^ of 1 M NaCl, shaken for two hours, removed from the shaker, and left for 24 h to allow the process of ion-exchange of sodium ions for ammonium ions. After this time the samples were centrifuged for 20 min at 14,000 rpm and then the solution was decanted from the sediment and transferred to a 25 cm^3^ flask. This process was repeated twice more, each time collecting the desorbent solution in the flask, which was finally made up to the mark.

For each sample, three weighings of 100 ± 1 mg were prepared initially. The CEC volume was presented as the average of the three analyses with a single standard deviation value. 

In the solution collected in the flask, the concentration of NH_4_ was determined using the Nessler method. NH_4_ reacts with Nessler’s reagent (K_2_HgI_4_) in the presence of sodium and potassium tartrate (Seignett salt) to give a colored complex. The intensity was determined on a Hitachi U-1800 UV-vis spectrophotometer at λ = 510 nm.

#### 2.4.3. Determination of the Anion-Exchange Capacity (AEC) by the Phosphate Method

The anion exchange capacity (AEC) was determined by the phosphate method: 100 mg of sample was placed in a polypropylene tube and 5 cm^3^ of phosphate buffer (KH_2_PO_4_ in K_2_HPO_4_) with a PO_4_ concentration of 0.04 M/dm^3^ was added. The suspension was shaken for 6 h maintaining the pH at 4.0. Then the tubes with the samples were centrifuged, the solution was poured off the sediment, and the shaking with the buffer was repeated twice. After the third shaking, the sample was rinsed three times with ethanol and dried to air-dryness. After drying, the sample was flooded with 5 cm^3^ of 1 M KNO_3_ with pH = 4.0 and shaken for 4 h. After centrifugation, the solution was transferred from the precipitate to a 25 cm^3^ flask. The shaking process with 1 M KNO_3_ was repeated twice more. After completing the mark with redistilled water, the concentration of desorbed PO_4_ ions was determined. The analysis was performed calorimetrically using the molybdovanadophosphoric acid method on a spectrophotometer. All determinations for each sample were made in triplicate. The result obtained is the average of four determinations.

### 2.5. Determination of the Sorption Capacity of Unmodified and Modified Samples towards NO_3_, PO_4_, SO_4_ Ions

Aqueous solutions of KNO_3_, KH_2_PO_4_, and K_2_SO_4_ of equal concentration equal to 0.1 M were prepared for the sorption study. To 0.5 g of each sample (crude, modified with HDTMA and modified with Ca^2+^ ions), 25 mL of each solution was added. The mixtures were shaken for 24 h, centrifuged for 5 min at 4500 rpm, and decanted the solution of the precipitate into a 100 cm^3^ flask. The samples were rinsed with redistilled water and the eluent was transferred to the same volumetric flasks as before. The amount of adsorbed ions was determined from the concentrations in the initial solutions and after sorption. The analyses were performed by UV–Vis spectrophotometry with a Hitachi U-1800 spectrophotometer at the wavelength λ = 510 nm.

### 2.6. Determination of the Phytoavailability of Adsorbed Ions NO_3_, PO_4_, SO_4_

Phytoavailability was verified according to the US EPA, Toxicity Characteristic Leaching Procedure, Appendix 1 Federal Register 51:216, 1986. To each sample containing adsorbed NO_3_, PO_4_, SO_4_ ions, 5 mL of extraction solution (0.02 M EDTA and 1 M CH_3_COONH_4_, pH 7) was added. The mixtures were shaken for 1 h, centrifuged for 5 min at 4500 rpm, and the solution was decanted from the precipitate. The resulting solutions were analyzed for the presence of individual ions by UV–Vis spectrophotometry (Hitachi U-1800, Hitachi Science & Technology, Berkshire, UK) at the wavelength λ = 510 nm.

## 3. Results and Discussion

### 3.1. Characteristics of RM and AS Materials

#### 3.1.1. XRD Analysis

**Figure 5:**
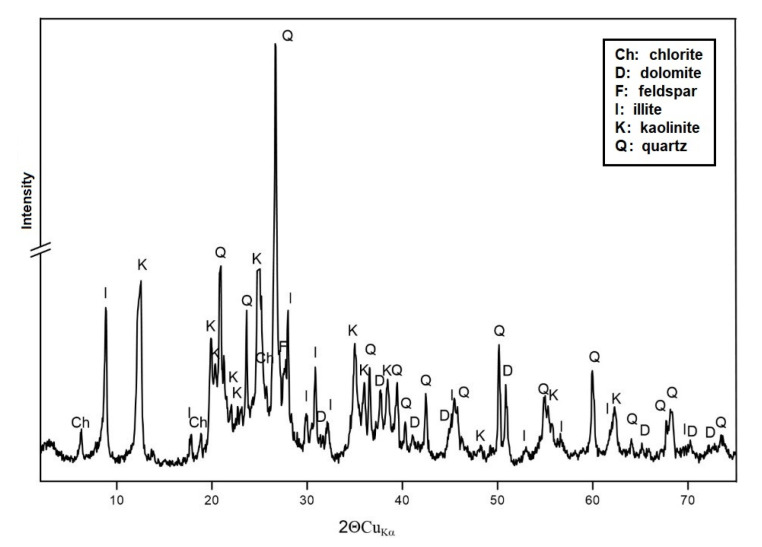
Diffractogram of sample RM.

Figure 5 shows the results of the X-ray analysis obtained for the RM sample.

The mineral composition of sample RM is dominated by quartz, kaolinite, and illite. They are accompanied by dolomite, chlorite, and feldspar.

**Figure 6:**
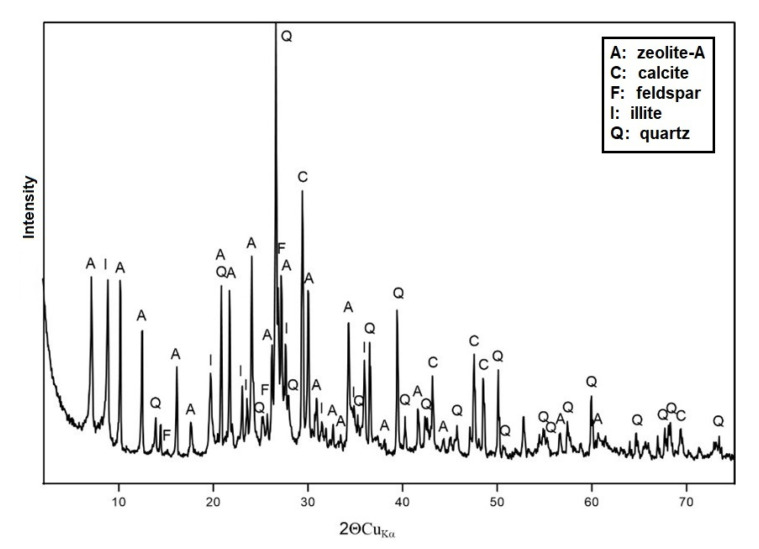
Diffractogram of sample AS.

Figure 6 shows the mineralogical analysis, as measured by an X-ray diffractometer, for sample AS.

The diffractogram obtained shows very high peaks arising from phases such as quartz and calcite. However, the overall diffractometric analysis of the AS material is dominated by peaks originating from zeolite-A. Additionally, phases such as feldspar and ilite were included in the analysis of the AS material.

#### 3.1.2. Texture and Morphology (N_2_ Adsorption/Desorption and SEM)

**Figure 7:**
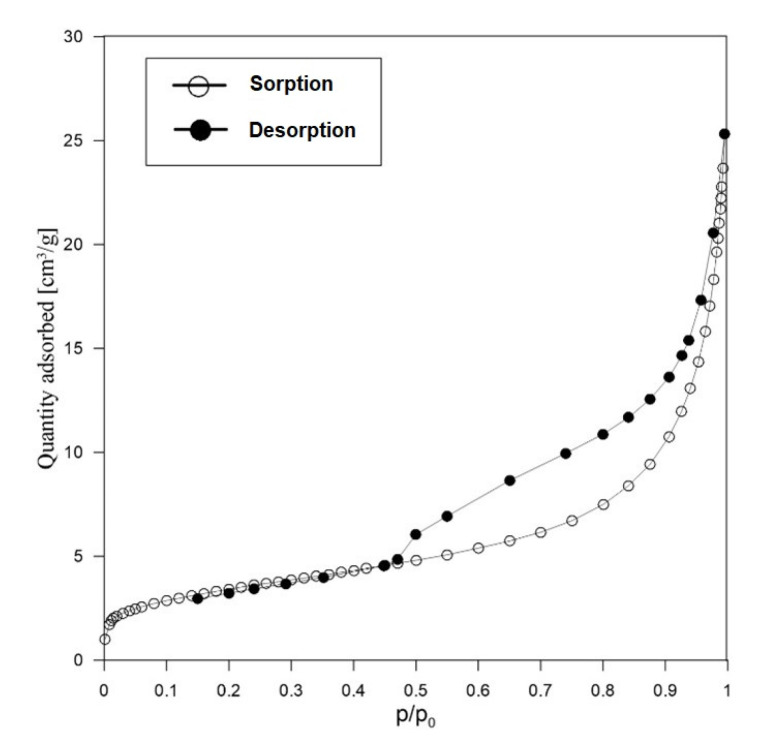
Isotherm of N_2_ sorption/desorption on RM sample after calcination.

Figure 7 shows the sorption/desorption isotherm obtained for sample RM after calcination. 

The isotherm presented represents type IV isotherm, characteristic of mesoporous materials. The results presented in Table 3 show that the proportion of micropores in this sample is 14%, mesopores 57%, and macropores 29%.

**Figure 8:**
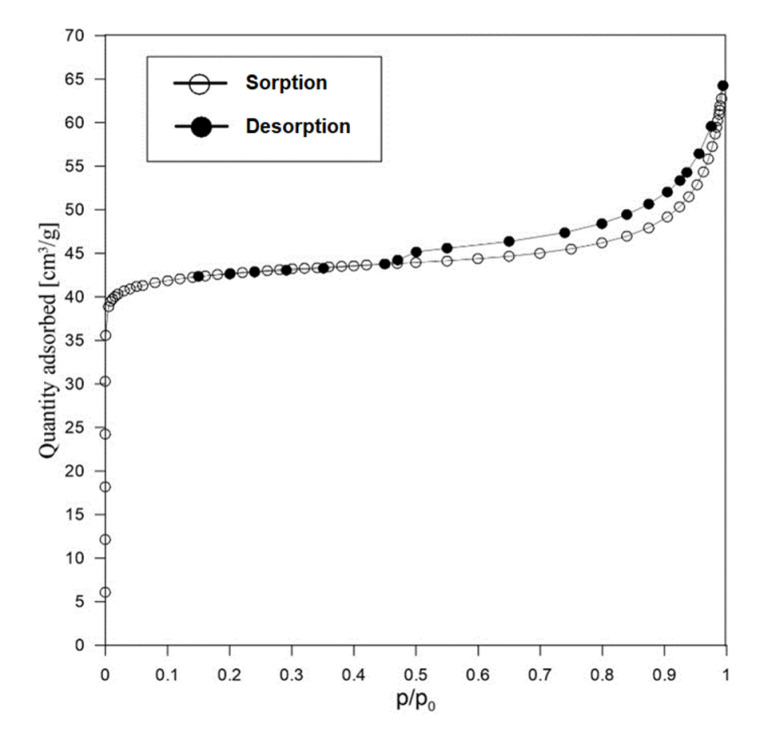
N_2_ sorption/desorption isotherm in AS sample.

Figure 8 shows the sorption/desorption isotherm obtained for sample AS.

The isotherm presented represents a type I isotherm, characteristic of microporous materials. The results presented in Table 3 (based on [46,49]) show that the proportion of micropores in this sample is 68%. The proportion of mesopores is 20% and macropores 12%.

The SBET surface results show that the samples after AS synthesis show more than 10 times the surface value (increase from 12 m^2^/g to 172.0 m^2^/g). The porosity distribution has also changed and in the samples after synthesis, as much as 68% of the micropores is observed in relation to 14% of the micropores for the reference sample. The increase in micropores increases the possibility of adsorption, especially of cations, which is shown by the comparison of this parameter for the tested sample (Table 5).

**Figure 9:**
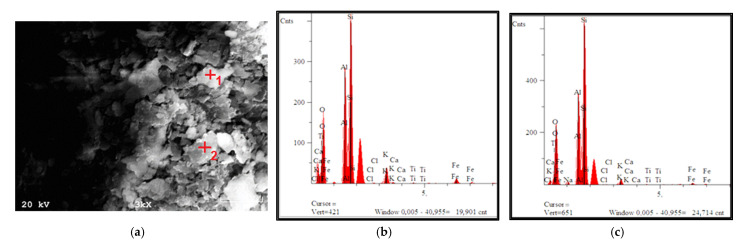
Particle morphology: (**a**) material after calcination (before synthesis and modification); (**b**) qualitative chemical analysis at point 1; (**c**) qualitative chemical analysis at point 2.

Figure 9 shows the particle morphology of the base material after calcination with a qualitative analysis of the chemical composition.

Both the shape and size were irregular. The analysis of the chemical composition showed the presence of mainly elements such as Si, Al, O, K, and Fe.

**Figure 10:**
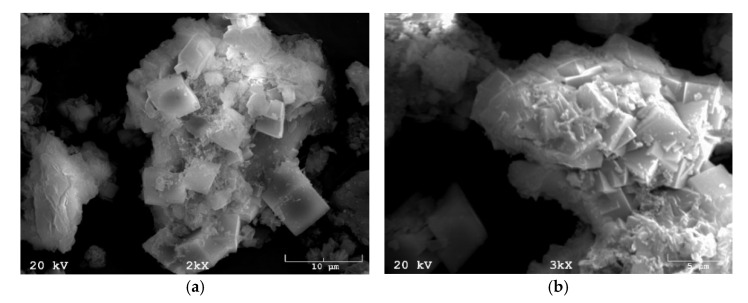
Morphology of the zeolite fractions obtained from the coal shale synthesis process: (**a**) at 2000× magnification; (**b**) at a magnification of 3000×.

Figure 10 shows the morphology of the zeolite fractions obtained from the coal shale synthesis process.

The transformation of the material used to produce zeolites can be seen in Figure 10. Crystals of approximately cubic shape can be seen, clustered in cascades. The size of a single crystal oscillates around 5 µm.

### 3.2. Modification Results for M-Ca and M-HDTMA Samples

Table 4 shows the Ca^2+^ content (extracted with ammonium chloride) of the samples before modification (AS) and after modification (M-Ca).

The increase in Ca concentration in the M-Ca sample indicates an effective modification of the sample, i.e., an increased concentration of Ca^2+^ ions introduced into the ion-exchange positions of the Ca-modified zeolite. For the unmodified sample designated AS, the amount of Ca^2+^ that originally occupied the ion exchange positions was 56% lower compared to the sample after Ca modification designated M-Ca.

The cation exchange capacity of CEC determined by HDTMA sorption for the sample was 13.57 meq/100 g.

### 3.3. CEC and AEC

Table 5 presents a comparison of the CEC and AEC values of the AS sample and other zeolite materials, natural and obtained by different synthesis methods. 

**Table 5:** CEC and AEC for sample AS and other natural zeolite materials, which were obtained by different synthesis methods.

Sample	CEC (meq/100 g)	AEC (meq/100 g)	Raw Material	Synthesis Conditions			References
				Solids to Solution/ Activator Ratio	Activator	Temperature/ TIME	
AS	138.93	19.86	Calcined Coal Shale	1.5 g/5 g NaOH	NaOH	80 °C/24 h	In article
FBB Met. N	79.57	84.26	Fluidized ash	10 g/0.1 dm^3^ NaOH	3 M NaOH	21 °C/30 days	[49]
FBB Met. F	104.32	83.24	Fluidized ash	S/NaOH—0.83	NaOH	100 °C/24 h (activation) 60 °C/24 h (crystallization).	[49]
KL SŁ WF	68.73	17.38	Slovak clinoptilolite	no treatment	no treatment	no treatment	[50]
AA01A5M160-2H	18.95	84.90	Fly ash fluidized bed “Green Block”, forest + sunflower biomass	2.5 g/10 mL	2 M NaOH	140 °C (autoclave)—24 h	[50]
AA04A5M160-2H	110.78	54.87	Fly ash from co-burning, 20% of the biomass including 20% chips, 80% sunflower + straw	2.5 g/10 mL	2 M NaOH	140 °C (autoclave)—24 h	[50]

The AS sample has one of the highest CEC values among the materials presented in Table 5, which indicates its high cation exchange capacity towards NH_4_. The AEC value obtained for the AS sample is similar to that obtained for natural zeolite—Slovak clinoptilolite. The value of cation exchange capacity CEC is the highest for the analyzed AS material. This parameter is higher for the investigated sample than for natural zeolites, e.g., clinoptilolite, or synthetic materials obtained by synthesis of zeolites from, e.g., fluidized bed ash or ash from biomass co-firing.

### 3.4. Results of Sorption, Desorption, and Phytoavailability of NO_3_, PO_4_, SO_4_

The number of anions adsorbed by a specific type of material is shown in Table 6.

The AS sample and the M–Ca sample had similar NO_3_ concentrations. The NO_3_ sorption of the M–HDTMA sample was more than 6 times lower compared to AS and M–Ca and amounted to 31.6 mmol/kg. The highest PO_4_ sorption value was obtained in the AS sample (about 870 mmol/kg), while the lowest value was achieved in the M–Ca sample (about 106 mmol/kg). The PO_4_ sorption value for the M–HDTMA sample in this case was about 560 mmol/kg. The M–Ca sample (about 1514 mmol/kg) achieved the highest value of absorbed SO_4_. Slightly lower values were obtained for the M–HDTMA sample, of approximately 1166 mmol/kg, however, the AS sample had the lowest NO_4_ sorption results.

The results of the phytoavailability experiment are presented in Table 7.

The highest NO_3_ desorption value was obtained in the AS sample (about 200 mmol/kg). A slightly lower value was achieved by sample M-Ca (about 180 mmol/kg). The lowest desorption value was obtained for sample M-HDTMA (about 31 mmol/kg). Also for the desorption of PO_4_, the highest value was obtained in the AS sample (about 788 mmol/kg). The lowest value was obtained in sample M-Ca—about 94 mmol/kg. The desorption value for M-HDTMA was about 526 mmol/kg. The SO_4_ desorption values for the AS and M-HDTMA samples were similar to each other, approximately 752 mmol/kg for AS and approximately 742 mmol/kg for M-HDTMA. The highest SO_4_ desorption value was obtained in the M-Ca sample (about 910 mmol/kg).

Table 8 shows the percentage loss of adsorbed anions from the samples that occurred in the phytoavailability experiment. This is a measure of the availability of anions to plants. The higher the percentage, the greater the susceptibility of the anion to desorption and availability to plants [51].

The highest desorption percentage for NO_3_ ions was achieved by sample M-HDTMA—97%, a slightly lower value was obtained by sample AS-93%. The M-Ca sample obtained a value of 83%. The desorption of PO_4_ ions for AS and M-Ca samples remained at a similar level-about 90%. M-HDTMA obtained the highest desorption percentage in this case, amounting to 94%. For the desorption of SO_4_ ions, samples M-Ca and M-HDTMA obtained similar values—about 60%. The desorption of SO_4_ for the AS sample was almost 90%.

## 4. Conclusions

The aim of this study was to modify the surface of synthetic zeolite materials with Ca and HDTMA compounds and to determine their phytoavailability by comparing CEC and AEC parameters. Zeolite materials, previously calcined, even without modification with Ca and HDTMA additives, showed very high sorption values of nitrates, phosphates, and sulphates during the study. Modification of the material with HDTMA and Ca surfactant resulted in improved sorption of sulphate compounds compared to the unmodified material.

Furthermore, modification with HDTMA surfactant and Ca showed that:

- for nitrogen and phosphorus compounds, M-HDTMA samples had the best phytoavailability (97%),
- for the sample M-Ca, the phytoavailability oscillated in the limits of 83% (for nitrogen compounds) and 89% (for phosphorus compounds), and
- the phytoavailability of sulphates for the modified samples (M-Ca and M-HDTMA) oscillated around 60%.

The material without modification (AS) obtained the highest value of phytoavailability of sulphates (88%). Moreover, in comparison to all types of analyzed materials (M-Ca and M-HDTMA), the percentage desorption of NO_3_, PO_4_, and SO_4_ was similar (90%). This may indicate the good ability of AS to release mineral compounds and its potential use as a fertilizer additive.

## Figures and Tables

**Table 1:** Petrographic composition of the material used in the tests.

Name of Mine	Petrographic Composition					Loss of Calcination
	Clay	Siltstones	Sandstones	Clay Siderites	Coal	
	(%)	(%)	(%)	(%)	(%)	(%)
KWK Rydułtowy—Anna Ruch Rydułtowy	46	26	28	0	0	3.3

**Table 2:** Description of samples according to their preparation.

Name of Sample			
RM	AS	M-Ca	M-HDTMA
Raw material	After synthesis	Modified Ca	Modified HDTMA

**Table 3:** Results of the porous texture analysis of samples [45,48].

Sample	Porous Texture Parameters				
	SBET (m^2^/g)	$V_{tot}^{0.99}\;({\mathrm{cm}}^{3}/g)$	$V_{mik}^{DR}\;({\mathrm{cm}}^{3}/g)$	$V_{mez}^{BJH}\;({\mathrm{cm}}^{3}/g)$	$V_{mak}\;({\mathrm{cm}}^{3}/g)$
RM (reference)	12.0	0.035	0.005	0.020	0.010
AS	172.0	0.096	0.065	0.019	0.008

SBET (m^2^/g)—specific surface according to Brunauer–Emmett–Teller (BET) theory; $V_{tot}^{0.99}$ (cm^3^/g)—total specific volume of pores for a relative pressure p/p_0_ = 0.99; $V_{mik}^{DR}$ (cm^3^/g)—the volume of micropores (pores with widths under 2 nm) according to the Dubinin–Radushkevich method; $V_{mez}^{BJH}$ (cm^3^/g)—the volume of mesopores (pores with a width greater than 2 nm and less than 50 nm) according to the Barrett–Joyner–Halve (BJH) method; $V_{mak}$ (cm^3^/g)—the volume of macropores (pores wider than 50 nm).

**Table 4:** Ca content extracted with 1 M NH_4_Cl.

Sample	AS	M-Ca
Concentration of Ca (meq/100 g)	154.2	273.4

**Table 6:** Sorption values of NO_3_, PO_4_, and SO_4_ in raw and modified samples.

	Sorption (mmol/kg)		
Sample	NO_3_	PO_4_	SO_4_
AS	213.6	871.6	854.1
M-Ca	218.7	105.9	1513.8
M-HDTMA	31.6	560.1	1165.6

**Table 7:** Desorption results of NO_3_, PO_4_, and SO_4_ from raw and modified samples.

	Desorption (mmol/kg)		
Sample	NO_3_	PO_4_	SO_4_
AS	199.5	788.4	752.5
M-Ca	181.2	93.9	910.5
M-HDTMA	30.8	526.3	741.6

**Table 8:** Percentage desorption (phytoavailability) of NO_3_, PO_4_, and SO_4_ from raw and modified samples.

	Desorption (%)		
Sample	NO_3_	PO_4_	SO_4_
AS	93	90	88
M-Ca	83	89	60
M-HDTMA	97	94	63

## Data Availability

Not applicable.
